# Balance, Gait, Functionality and Fall Occurrence in Adults and Older Adults with Type 2 Diabetes Mellitus and Associated Peripheral Neuropathy

**DOI:** 10.3390/clinpract14050161

**Published:** 2024-09-28

**Authors:** Natália Maria Bezerra Tavares, Jonathânya Marques Silva, Mayra Darlene Morato da Silva, Letícia Danielly Tenório Silva, Jackson Nascimento de Souza, Lucas Ithamar, Maria Cristina Falcão Raposo, Renato S. Melo

**Affiliations:** 1 Department of Physical Therapy, Faculdade de Integração do Sertão (FIS), Serra Talhada 56909-205, PE, Brazil; 2 Department of Physical Therapy, Universidade Federal de Pernambuco (UFPE), Recife 50670-901, PE, Brazil; 3 Department of Statistics, Universidade Federal de Pernambuco (UFPE), Recife 50670-901, PE, Brazil; 4 Department of Medicine, Faculdade de Medicina do Sertão (FMS), Arcoverde 56512-670, PE, Brazil

**Keywords:** accidental falls, ageing, aging, diabetic neuropathies, motor skills disorders, perception, postural balance, sense organs, urinary incontinence, walking

## Abstract

Background: Body balance is regulated by sensory information from the vestibular, visual and somatosensory systems, and changes in one or more of these sensory systems can trigger balance disorders. Individuals with type 2 Diabetes Mellitus (DM2) often present peripheral neuropathy, a condition that alters foot sensory information and can negatively influence balance and gait performance of these subjects. Objective: To evaluate and compare balance, gait, functionality and the occurrence of falls between individuals with and without a clinical diagnosis of DM2 with associated peripheral neuropathy. Methods: Cross-sectional study, which evaluated seventy individuals, thirty-five with and thirty-five without a clinical diagnosis of DM2, of both sexes and age range between 50 and 85 years, who were recruited from Basic Health Units of Serra Talhada, Pernambuco state, Brazil. The volunteers’ balance was analyzed using the Berg Balance Scale, gait-related functional tasks were measured using the Dynamic Gait Index, functional mobility was assessed using the Timed Up and Go test and functionality was assessed using the Katz Index. The occurrence of falls was recorded by the volunteers’ self-report. Results: Individuals with DM2 demonstrated the worst performance in balance (*p* = 0.000) and in gait-related functional tasks (*p* = 0.000), slower functional mobility (*p* = 0.000) and worse functionality (*p* = 0.016) compared to the group without DM2, demonstrating significant differences for all analyzed outcomes. A greater occurrence of falls was observed in individuals with DM2, compared to those without the disease (*p* = 0.019). Conclusion: Individuals with DM2 demonstrated worse performance on balance, gait-related functional tasks, slower functional mobility and worse functionality compared to those without the disease. Individuals with DM2 had the highest occurrence of falls in this study.

## 1. Introduction

Diabetes Mellitus is a syndrome with multiple etiologies, resulting from the lack of insulin and/or the inability of this hormone to properly exert its effects, which can lead to the development of associated diseases and serious complications such as retinopathies and nephropathies. Furthermore, the mobility and functionality of the individuals with Diabetes Mellitus can also be impaired due to peripheral neuropathy and loss of joint mobility and muscle strength [1,2,3,4].

Peripheral neuropathies are characterized by the progressive loss of nerve fibers of the somatic nervous system. The simple and internationally accepted definition of diabetic neuropathies is the presence of signs and/or symptoms of peripheral nervous dysfunction in patients with Diabetes, after excluding other possible causes [5]. Peripheral neuropathy is one of the most common complications resulting from Diabetes Mellitus, constituting one of the biggest causes of neuropathy in the world, in addition to being the most significant cause of morbidity and mortality. This complication refers to the set of clinical syndromes that affect the peripheral sensory and motor nervous system, being the causal agent, that is, what initiates the pathophysiological process, leading to changes/reduction in peripheral sensitivity, ulcerations and amputations in more severe cases [5,6].

This sensitivity reduction is one of the main aspects that contribute to the reduction of sensory input for the regulation of the motor and postural control system and, therefore, for the regulation of body balance [7], which can generate changes in body stability and gait, such as such as lower cadence, higher steps, and less acceleration, as well as slowness in correcting motor errors, such as, for example, when it is necessary to overcome an obstacle. Thus, it can be inferred that individuals with peripheral neuropathy are more likely to suffer episodes of falls and also have difficulty going up and down stairs or walking through busy streets and on uneven floors [8,9,10,11].

Evidence shows that subjects with type 2 Diabetes Mellitus (DM2) have at least one functional disability and difficulties in walking 400 m, doing household chores or climbing stairs when compared to those without DM2. Given this evidence of incapacity and worse performance when performing everyday tasks, investigations whose objectives are to compare physical–functional performance between individuals with and without DM2 through tests that evaluate multiple domains of the physical–functional function become relevant, demonstrating how these domains are currently in these individuals so that intervention measures can be designed in the future by trained professionals [11,12,13,14].

Given the likelihood of the individuals with DM2 presenting changes in plantar sensitivity and, as the somatosensory system is one of the responsible systems by the balance and postural control [15], this population may present changes in balance compared to those without the disease due to hypoesthesia plantar [16]. Some studies involving individuals with DM2 have assessed balance without considering diabetic peripheral neuropathy. The most serious issue is that some investigations have aggregated subjects with and without diabetic peripheral neuropathy in the same sample and results, contributing to biased and unreliable results.

Given the importance of body balance for functional activities and activities of daily living, such as gait, this investigation chose to investigate not only balance but also the functional outcomes of individuals with DM2, such as gait and functionality, and also the occurrence of falls in this population. Given the above, the primary objective of this study was to evaluate and compare the performance of balance, gait, functionality and the occurrence of falls in individuals with and without DM2. The secondary objective was to compare the balance, gait and functionality between groups, according to sex and age group.

## 2. Materials and Methods

This cross-sectional study was developed in accordance with the recommendations of the Strengthening the Reporting of Observational Studies in Epidemiology (STROBE) [17,18,19] guidelines for the development of observational studies.

### 2.1. Sample

The sample of this study was recruited from the Basic Health Units (BHUs) of Serra Talhada, Pernambuco state, Brazil. The inclusion criteria for this study were individuals of both sexes, aged between 50 and 85 years, with or without a clinical diagnosis of DM2 duly diagnosed through a medical report from the BHU (it was a purely clinical diagnosis), presenting a good general state of health to carry out the tests, and not use walking aids. In addition to the above criteria, individuals with DM2 should have their disease under control and have peripheral neuropathy confirmed by the physician.

The exclusion criteria were having amputations in any region of the lower or upper limbs and individuals with open ulcers; with congenital/acquired deformities; who were using medications that reduce cognition; with a history of orthopedic, rheumatological and neurological diseases; and with report of recurrent dizziness or vertigo.

### 2.2. Data Collection

Data collection took place in the BHUs, where the individuals of this study were seen weekly by physicians and other health professionals. They were invited by the researchers to participate in this research and those who agreed signed an informed consent form. The assessments were carried out in a private room at the BHUs, kindly provided by the directors of the units to collect data for this study.

The evaluation began with the collection of personal data from each volunteer using the evaluation form, which contained the following information: name, age, sex, height, weight, BMI, presence of high blood pressure and whether the volunteers had had episodes of falls in the last six months.

The researchers individually explained to the volunteers all the procedures and how the assessments of the balance, gait and functionality would take place, and when there were no more doubts, the evaluations began.

We began the evaluations by assessing balance using the Brazilian version of the Berg balance scale [20], validated for the Brazilian population, which assesses the volunteer’s functional balance based on their functional performance in 14 items: from sitting to standing, standing without support, sitting without back support, standing to sitting, performing transfers, standing without support and with eyes closed, standing without support with feet together, reaching forward with arms extended while remaining standing, picking up an object on the floor from a standing position, standing and looking behind shoulders, turning 360°, alternating feet on steps without support, standing with one foot in front of the other and standing supported only on one of the legs.

This scale presents a score for each item that ranges from 0 to 4 points, where 0 (zero) means that the volunteer was unable to perform the item, and 4 (four) indicates that the item was performed with satisfactory performance. The volunteer is evaluated according to his or her total performance on the scale, receiving a score according to his or her performance; the maximum value of the scale is 56 points, and the higher the score obtained by the volunteer on the scale, the better his or her balance [20].

Gait-related functional tasks were assessed using the Dynamic Gait Index (DGI), also validated for the Brazilian population [21]. The DGI consists of eight tasks, assessing gait in walking on a flat surface, changes in the gait speed, gait with horizontal and vertical movements of the head, walking and turning on own axis, walking and stepping over obstacles, walking around and around obstacles and going up and down steps. The DGI has four scores, which must be used in each of the eight tasks, according to the volunteers’ performance, ranging between 0 and 3, with 0 (zero) meaning severe performance and 3 (three) adequate performance, according to the test. The DGI has a total score between 0 and 24 points, so the higher the score obtained by the individuals, the better their performance in gait-related functional tasks.

Then, the functional mobility of the volunteers was assessed using the Timed Up and Go (TUG) test, recording using a digital stopwatch the time spent in seconds that the volunteers took to get up and leave an initial chair without support of their arms, walk towards another chair located at a distance of 3 m, go around that chair and return and sit in the initial chair, carrying out this entire route at their normal speed and with bare feet [22,23].

To finalize the evaluations, the functionality was analyzed using the Brazilian version of the Katz Index [24], an instrument that contains data on functional independence in relation to the home on the following aspects: bathing, dressing, eating, going to the bathroom, transfers and continence. For each of these items, a score between 0 and 1 point is given, with 0 (zero) when the individual reports dependence on performing the item, half a point when receiving assistance to perform the item and 1 (one) point is assigned when the volunteer performs the item independently. Thus, the individuals’ functionality can be between 0 (worst performance) and 6 points (best performance) according to the functionality reported by the volunteer [24].

To assess the occurrence/history of falls, the volunteers were asked whether, in the last six months, they had experienced episodes of falls, with the only answer being yes or no. Reports of falls were checked with the volunteers’ medical records at the HBUs and with reports from the family members or caregivers of the older adult volunteers.

### 2.3. Statistical Analysis

Data regarding the balance, gait and functionality performance of each volunteer in this study were recorded on the instruments and then included in the database. Data entry into Microsoft Excel 2010 was performed by two independent researchers (double data entry) to avoid typing errors and ensure greater reliability of the findings of this study [25,26,27]. Once this was performed, the data were transferred to the Statistical Package for the Social Sciences (SPSS), version 20.0, where all analyses were carried out, adopting a statistical significance level of 5%.

To test the normality of quantitative variables, the Kolmogorov–Smirnov test was used. When comparing means between two groups, the Mann–Whitney test was used for cases of non-normality or the student’s *t*-test for cases compatible with normal distribution. For dichotomous variables, Pearson’s chi-square or Fisher’s exact test of independence was used when necessary.

After the bivariate analysis of the possible explanatory factors, for each of the outcomes analyzed by group, multiple linear regression models were adjusted to explain the aforementioned outcomes according to age and BMI. The models were evaluated using the t-test for significance of the parameters, the *p*-value of the ANOVA, as well as the coefficient of determination r^2^ measured in percentage.

This study was evaluated and approved by the Research Ethics Committee of the Faculdade de Integração do Sertão—FIS on 19 September 2019, according to the following CAAE number: 14556919.9.0000.8267 and register number: 3.586.358.

## 3. Results

The sample of this research was made up of seventy volunteers, thirty-five with and thirty-five without DM2, being a convenience sample.

The characterization of the volunteers is described in Table 1. The group with DM2 showed worse performance in balance, gait and functionality, presenting significant differences, compared to the group without the disease, as shown in Table 2.

Regarding sex, women demonstrated worse performance when comparing groups with and without DM2, showing significant differences for all outcomes. In men, significant differences were observed for balance and gait performance, and the group with DM2 always presented worse performance, as shown in Table 3.

Table 4 presents the distribution of the sample’s performance according to the stratified age groups, and the group with DM2 presented worse performances in all age groups for all analyzed outcomes. However, significant differences between groups were observed only in the balance and gait assessments, in all age groups. The occurrence of falls was higher in the group with DM2 compared to the group without the disease, demonstrating significant differences (*p* = 0.019) according to Table 5.

The main results of the adjusted multiple linear regression models are presented in Table 6 below, from which it is possible to conclude that more than 97% of the variation in each outcome can be explained by the variation in age and BMI, except for the DGI data, with the group with DM2 accounting for 46.2% and the group without DM2 accounting for only 16.9%.

## 4. Discussion

It was observed that subjects with DM2 presented worse balance compared to those without the disease, in relation to groups and sexes, with significant differences. This can be justified by the presence of the peripheral neuropathy in the DM2 group of this study as peripheral neuropathy has been documented as a risk factor for increased balance disorders in patients diagnosed with DM2 [28] and as a condition often found in these individuals [29,30,31].

Sensory information from the plantar region of the feet is important for maintaining an upright posture and balance [32]. In the absence of sensory information from the plantar region, as is common in peripheral neuropathies, mechanoreceptors and ankle movements are impaired. Peripheral neuropathies cause loss of somatosensation, originating from mechanoreceptors located in the skin, and individuals who have this condition are unable to remain standing without the assistance of external assistive devices, such as the use of canes and walkers, for example [32].

Therefore, without information on plantar pressure differences (sensory information from the soles of the feet), the vestibular reflexes are unable to maintain postural control and balance [32,33], triggering changes in ankle strategy [34] and, consequently, balance deficits such as those identified in the volunteers with DM2 in this study. These findings also corroborate the findings of other investigations that found similar results, using different instruments, concluding that individuals with DM2 had balance disorders [35,36,37,38], and these disorders can predict changes in the gait in this population [38].

Individuals with DM2 in this study also showed changes in gait-related functional tasks and slower functional mobility when compared to those without DM2, in relation to groups and sexes, demonstrating significant differences.

The changes in the gait observed in individuals with DM2 in this study may be related to sensory loss in the plantar region of the foot, loss of protective sensation in the feet and weakness of the intrinsic muscles of the foot [39]. The presence of peripheral neuropathy has been the strongest independent risk factor for gait abnormalities in individuals with DM2 [40], and it is worth highlighting that all subjects with DM2 in this study had peripheral neuropathy diagnosed by doctors, as it was one of the criteria for inclusion in the group with DM2, and this would justify the changes in the gait found in individuals with DM2 in this study.

In addition to the presence of peripheral neuropathy, changes in the kinetic and kinematic gait characteristics of individuals with DM2, found by other investigations, could also justify the changes in the gait-related functional tasks and slower functional mobility found on subjects with DM2 in this study. The main changes reported in the literature include slower gait speed, reduced cadence and step length, reduced stride speed and increased double support phase in gait [41,42,43,44,45]. In addition, elevated plantar pressure, reduced movements in the ankle joint, increased stretching of the triceps surae muscles during terminal support and a prolonged increase in hip extension during the mid-support phase of the gait have also been reported [45,46,47].

All of these conditions described above may have contributed to the subjects with DM2 in this study demonstrating worse gait performance in the Timed Up and Go test and DGI as observed. These findings corroborate other studies, which found similar results, even using other instruments, concluding that subjects with DM2 present changes in the gait [48,49,50,51].

Furthermore, women performed worse in all outcomes analyzed. Women show impairments of balance when simultaneously deprived of visual and somatosensory inputs or during a backwards destabilization [52]. Which also could justify the difficulties in the walking and functionality observed in this study. Since there is little evidence for a central nervous system source for such sex differences, biomechanical origins (e.g., dorsiflexion strength and range of motion) are a more likely cause for these clinical differences [52].

It is worth mentioning that we did not find evidence published in the literature that analyzed gait performance of subjects with DM2 using the DGI. The DGI is an instrument that evaluates gait-related functional tasks in dual-task conditions, widely reliable and used, and validated for the Brazilian population, which is an important contribution of this study for the gait outcome.

Regarding functionality, there was a significant difference in relation to the groups and the female sex, always with the group with DM2 demonstrating the worst functionality.

The limited functionality in the DM2 group in this study is exclusively related to one of the items in the Katz index, as some of the female volunteers reported that they had urinary incontinence, which made their functionality difficult and reduced their score in the functionality assessment. This finding is in line with recent investigations, which found a significant correlation between DM2 and urinary incontinence in women [53,54,55,56], justifying our findings in relation to functionality.

When the sample was divided into age groups, individuals with DM2 demonstrated the worst clinical performance in all outcomes analyzed in all age groups. Those with DM2 and aged ≥70 years presented the worst performance among all age groups and significant differences for balance and gait outcomes. Aging alone also showed a relationship with balance performance, with gait-related functional tasks and with functionality in the group with DM2 according to the adjusted linear regression model. It is known that aging causes changes in all systems of the human body, and the same occurs in the systems responsible for regulating postural control and balance. Evidence in the literature shows that balance and gait worsen and lead to falls with increasing age in healthy older adults [57,58,59,60,61,62,63], as can be seen in the balance and gait results of subjects without the disease in this study.

However, when added to the physiological changes of aging with the sensory changes caused by DM2, such as peripheral neuropathy, for example, the repercussions for balance and gait were much more serious in the group with DM2. Other investigations also found balance and gait disorders in older adults with DM2 [64,65,66] and concluded that this population has a 1.25-times higher risk of having balance and gait disorders [64] compared to their peers without the disease, making this population more susceptible to falls.

Individuals with DM2 had a higher occurrence of falls in the last six months when compared to those without the disease. This finding seems to come from changes in balance and gait observed in the sample since the literature shows that disturbances in balance and gait increase the fear of falling in people with DM2 [67,68,69]. Falling is an important outcome as it is the main cause of fatal and non-fatal injuries in older adults and the main cause of traumatic brain injury in the older population. In addition to traumatic brain injury and fractures, non-fatal falls result in reduced levels of physical activity, favoring the fear of falling and generating a condition of kinesiophobia and reduced functionality and quality of life in older adults after episodes of falls [70,71,72,73].

This demonstrates the importance of including individuals with DM2 in intervention programs to improve balance and gait and prevent episodes of falls in this population. There is evidence that multicomponent exercise therapy consisting of strength exercises and range of motion, balance, flexibility and gait training improves balance and gait in people with DM2 and associated peripheral neuropathy [74,75,76,77,78]. Furthermore, aquatic exercises have also been shown to be effective in improving balance and gait and in reducing the fear of falling in this population [79,80,81], demonstrating that individuals with DM2 can benefit from these exercise programs to achieve better functionality and quality of life.

This study identified that individuals with DM2 presented more changes in balance, gait and functionality compared to those without the disease. Such data are relevant as they provide evidence and highlight how much this population needs care and how much the professional physiotherapist can help these individuals with their needs, such as, for example, in the rehabilitation of the balance, gait and also urinary disorders.

We also highlight that the role of the physiotherapist for the diabetic population can extend to sensory guidelines and adaptations and usual care, referring to ulcers and their risks of amputations, which are already well known, and which limit functionality and reduce quality of life in patients with DM2 and associated peripheral neuropathy.

We mention, as limitations in this study, the low number of men in the sample, which was not estimated by sample calculation, which limits the generalization of its results. This can be accomplished by future studies on the topic. This investigation assessed community subjects assisted at BHUs, limiting these results to this population only.

## 5. Conclusions

Individuals with DM2 had worse balance, gait and functionality when compared to those without the disease.

The same occurred according to sex and age group in relation to balance and gait. However, functionality only showed significant differences with female sex, with no significant difference with any age group. Subjects with DM2 had the highest occurrence of falls in this study.

## Figures and Tables

**Table 1:** Characterization of the sample.

	With DM2 (n = 35)	Without DM2 (n = 35)	*p*-Value
	Mean ± SD	Mean ± SD	
**Age** (**y**)	63.7 ± 1.52	61.3 ± 1.59	0.278 ^a^
**Height** (**m**)	1.62 ± 0.10	1.64 ± 0.45	0.413 ^a^
**Weight** (**kg**)	65.4 ± 2.03	64.3 ± 1.82	0.729 ^a^
**BMI**	24.7 ± 0.58	24.0 ± 0.49	0.364 ^a^
**Diagnosis time** (**mth**)	38.6 ± 2.81	---	---
**Sexes:**	**n** (**%**)	**n** (**%**)	
Women	28 (78.1)	29 (85.3)	0.154 ^b^
Men	07 (21.9)	06 (14.7)	
**Peripheral neuropathy**			
Yes	35 (100)	0 (0)	---
No	0 (0)	35 (100)	

DM2: Type 2 Diabetes Mellitus; SD: Standard deviation; y: years; m: meters; kg: kilograms; mth: months; BMI: Body mass index; ^a^: Student’s *t* test; ^b^: Pearson’s chi-square test.

**Table 2:** Balance, gait and functionality of individuals with and without type 2 Diabetes Mellitus.

	With DM2 (n = 35)	Without DM2 (n = 35)	*p*-Value
	Mean ± SD	Mean ± SD	
**Balance**	45.1 ± 1.16	52.2 ± 0.84	**0.000 ^a^**
**Gait-related Functional Tasks**	16.1 ±0.49	21.5 ± 0.67	**0.000 ^a^**
**Functional Mobility**	19.5 ± 1.02	12.5 ± 0.93	**0.000 ^a^**
**Functionality**	5.69 ± 0.08	5.92 ± 0.05	**0.016 ^a^**

DM2: Type 2 Diabetes Mellitus; SD: Standard deviation; ^a^: Mann–Whitney test; Bold: *p*-value showed a significant difference.

**Table 3:** Balance, gait and functionality of individuals with and without type 2 Diabetes Mellitus, according to the sexes.

	Women			Men		
	With DM2 (n = 28)	Without DM2 (n = 29)	*p*-Value	With DM2 (n = 7)	Without DM2 (n = 6)	*p*-Value
	Mean ± SD	Mean ± SD		Mean ± SD	Mean ± SD	
**Balance**	45.4 ± 1.35	52.0 ± 0.95	**0.000 ^a^**	44.4 ± 2.45	53.5 ± 1.84	**0.009 ^a^**
**Gait-related Functional Tasks**	15.5 ± 0.56	21.5 ± 0.78	**0.000 ^a^**	18.1 ± 0.55	21.7 ± 1.31	**0.020 ^a^**
**Functional Mobility**	20.1 ± 1.27	12.7 ± 1.10	**0.000 ^a^**	17.5 ± 1.15	11.7 ± 0.52	**0.017 ^a^**
**Functionality**	5.64 ± 0.10	5.90 ± 0.06	**0.019 ^a^**	5.86 ± 0.14	6.00 ± 0.00	0.398 ^a^

DM2: Type 2 Diabetes Mellitus; SD: Standard deviation; ^a^: Mann–Whitney test; Bold: *p*-value showed a significant difference.

**Table 4:** Balance, gait and functionality of individuals with and without type 2 Diabetes Mellitus according to stratified age group.

	50–59 Years			60–69 Years			≥70 Years		
	With DM2 (n = 10)	Without DM2 (n = 14)	*p*-Value	With DM2 (n = 11)	Without DM2 (n = 13)	*p*-Value	With DM2 (n = 14)	Without DM2 (n = 8)	*p*-Value
	Mean ± SD	Mean ± SD		Mean ± SD	Mean ± SD		Mean ± SD	Mean ± SD	
**Balance**	46.7 ± 1.50	53.1 ± 1.10	**0.001 ^a^**	45.7 ± 2.82	51.8 ± 1.65	**0.029 ^a^**	44.0 ± 1.70	51.2 ± 1.49	**0.003 ^a^**
**Gait-related Functional Tasks**	17.4 ± 0.52	23.2 ± 0.61	**0.000 ^a^**	16.2 ± 0.57	20.5 ± 1.34	**0.005 ^a^**	14.7 ± 1.30	20.2 ± 1.03	**0.008 ^a^**
**Functional Mobility**	17.7 ± 1.22	10.8 ± 1.12	**0.006 ^a^**	19.5 ± 1.51	14.6 ± 1.64	**0.039 ^a^**	21.0 ± 2.40	16.0 ± 1.97	**0.008 ^a^**
**Functionality**	5.43 ± 0.20	5.90 ± 0.10	0.056 ^a^	5.90 ± 0.12	5.90 ± 0.10	0.722 ^a^	5.71 ± 0.12	5.70 ± 0.78	0.125 ^a^

DM2: Type 2 Diabetes Mellitus; SD: Standard deviation; ^a^: Mann–Whitney test; Bold: *p*-value showed a significant difference.

**Table 5:** Occurrence of falls among individuals with and without type 2 Diabetes Mellitus.

	With DM2 (n = 35)	Without DM2 (n = 35)	*p*-Value
	n (%)	n (%)	
**Yes**	19 (54)	9 (24.3)	**0.019 ^a^**
**No**	16 (46)	26 (75.7)	

DM2: Type 2 Diabetes Mellitus; ^a^: Pearson’s Chi-square test; Bold: *p*-value showed a significant difference.

**Table 6:** Results of the multiple linear regression model to explain the outcomes, by groups, according to age and BMI.

Y	Independent Variables	With DM2			Without DM2		
		Coefficient ß	*p*-Value (*)	r^2^ in %	Coefficient ß	*p*-Value (*)	r^2^ in %
**Balance**	Age	0.447	**0.002**	97.7	0.327	**0.001**	99.3
	BMI	0.494	0.157		1.205	** 0.000 **	
**Gait-related Functional Tasks** (**)	Age	0.142	** 0.037 **	46.2	0.170	** 0.001 **	16.9
	BMI	0.293	0.091		0.383	** 0.002 **	
**Functional Mobility**	Age	0.098	0.090	93.1	0.065	** 0.007 **	99.0
	BMI	0.308	** 0.041 **		0.281	** 0.000 **	
**Functionality**	Age	0.033	** 0.052 **	97.9	0.040	** 0.002 **	97.1
	BMI	0.128	** 0.005 **		0.133	** 0.000 **	

DM2: Type 2 Diabetes Mellitus; BMI: Body mass index; (*) *t*-test of significance of the parameters. (**) For these models, the ANOVA *p*-values were, respectively, 0.019 and 0.649 for the groups with and without DM2; Bold: *p*-value showed a significant difference.

## Data Availability

The original contributions presented in the study are included in the article, further inquiries can be directed to the corresponding author.
